# The Nutritional Content of Meal Images in Free-Living Conditions—Automatic Assessment with goFOOD^TM^

**DOI:** 10.3390/nu15173835

**Published:** 2023-09-02

**Authors:** Ioannis Papathanail, Lubnaa Abdur Rahman, Lorenzo Brigato, Natalie S. Bez, Maria F. Vasiloglou, Klazine van der Horst, Stavroula Mougiakakou

**Affiliations:** 1 ARTORG Center for Biomedical Engineering Research, University of Bern, 3008 Bern, Switzerland; ioannis.papathanail@unibe.ch (I.P.); lubnaa.abdurrahman@unibe.ch (L.A.R.); lorenzo.brigato@unibe.ch (L.B.); vasilogloumaria@gmail.com (M.F.V.); 2 School of Health Professions, Bern University of Applied Sciences, 3008 Bern, Switzerland; natalie.bez@bfh.ch (N.S.B.); klazine.vanderhorst@bfh.ch (K.v.d.H.)

**Keywords:** automatic dietary assessment, artificial intelligence, computer vision, food segmentation, food recognition, portion estimation, volume estimation, nutrient calculation

## Abstract

A healthy diet can help to prevent or manage many important conditions and diseases, particularly obesity, malnutrition, and diabetes. Recent advancements in artificial intelligence and smartphone technologies have enabled applications to conduct automatic nutritional assessment from meal images, providing a convenient, efficient, and accurate method for continuous diet evaluation. We now extend the goFOOD^TM^ automatic system to perform food segmentation, recognition, volume, as well as calorie and macro-nutrient estimation from single images that are captured by a smartphone. In order to assess our system’s performance, we conducted a feasibility study with 50 participants from Switzerland. We recorded their meals for one day and then dietitians carried out a 24 h recall. We retrospectively analysed the collected images to assess the nutritional content of the meals. By comparing our results with the dietitians’ estimations, we demonstrated that the newly introduced system has comparable energy and macronutrient estimation performance with the previous method; however, it only requires a single image instead of two. The system can be applied in a real-life scenarios, and it can be easily used to assess dietary intake. This system could help individuals gain a better understanding of their dietary consumption. Additionally, it could serve as a valuable resource for dietitians, and could contribute to nutritional research.

## 1. Introduction

Maintaining a healthy diet is crucial for overall well-being and is fundamental in preventing and managing various health conditions and diseases, such as cancer and diabetes. However, nutrition-related health issues are often undermined or overlooked, despite their significant impact on global health [1,2]. Additionally, it was reported that approximately 2.3 billion adults were affected by malnutrition alone, encompassing both under- and over-nutrition, in 2021 [3]. The consequences of these diet-related issues extend beyond individual health and have implications for global health. The burden they place on healthcare systems is substantial, straining resources while affecting the overall quality of healthcare services available, whereby nutrition-related conditions often extend hospital stays [4]. Therefore, if we are to effectively address health conditions related to diet and promote overall well-being, it is crucial to monitor dietary intake accurately.

While it is generally acknowledged that it is important to monitor and assess diet [5,6], there are multiple practical limitations to its implementation. Individuals may rely on various approaches to assess their diet, such as weighing their own meals or seeking assistance from dietitians or other healthcare professionals [7]. The latter approach primarily relies on the individuals’ food records, food frequency questionnaires (FFQs), or 24 h recalls [7]. Such methods often fail to provide quantitative or visual representations of the food intake and rely solely on the individuals’ descriptions, thus leading to subjective estimations and difficulties in accurately assessing portion sizes, thereby resulting in inaccurate evaluations and ineffective dietary management [8,9,10]. Moreover, the lack of nutritional literacy further exacerbates this challenge [11], which arises from individuals’ limited familiarity with foods and the units of measurement for their nutritional content, and it can potentially lead to less precise estimations. While reliance on professional experts for dietary assessments may be advocated, this can be time-consuming, burdensome, and prone to errors as it depends on available records and the individuals’ ability to accurately maintain detailed accounts of food consumption and to provide precise descriptions of food items. In addition, access to dietitians and healthcare services is not universal, particularly in countries or remote areas with limited healthcare infrastructure [12,13].

The need for more accessible, accurate, and less time-consuming dietary assessment is then evident [14]. Recent advancements in artificial intelligence (AI) and smartphone technology have provided new opportunities in this field. With the rise of mobile health and its widespread adoption, mobile apps have emerged as a promising solution for automating the process of dietary assessment [15,16]. These apps offer a comprehensive approach by incorporating food segmentation, food recognition, as well as estimations of portion size and nutritional content, and thus they may reduce the burden on individuals [17,18,19,20]. Numerous studies have compared smartphone app-based methods to conventional approaches and have emphasised positive outcomes, such as increased user satisfaction and preference for mobile-based approaches [21,22]. Nevertheless, it is important to note that most existing apps still heavily rely on manual or semi-automatic data entry, particularly for estimating portion sizes. Apps that have attempted to incorporate automatic portion estimation often necessitate phones equipped with depth sensors or the recording of lengthy videos or multiple frames from different angles, and thus introduce potential discrepancies in results based on different conditions [23]. Notably, errors in volume estimation have been predominantly observed in dietary apps, with reported errors reaching as high as 85% [24]. Nonetheless, the recent advancements in food image and video analysis have demonstrated the potential for fully automating the pipeline of dietary assessment [25,26].

In our previous publication, we demonstrated the effectiveness of goFOOD^TM^, an AI-based mobile application for automatic dietary assessment [27]. However, a significant limitation of the previous system was the requirement for users to capture two images or a short video, and this could be perceived as a burden. It was shown that people would rather take a single image while compromising on the accuracy of the final results instead of two images, which could provide results closer to the ground truth (GT) [28]. To overcome such challenges, we propose an enhanced system utilising a single image captured with a smartphone, and we compared this to an adaptation of the previous version [27]. While our previous study demonstrated the adequacy of AI-based dietary assessment in a controlled hospital setting [29], we now sought to assess its applicability in free living conditions.

## 2. Materials and Methods

In this manuscript, we conducted a study where we recruited participants from Switzerland to record short videos of the foods and beverages they consumed over the course of one day using the goFOOD^TM^Lite application, which is described in detail in Section 2.1. The participants were asked to complete a feedback questionnaire regarding their satisfaction with the goFOOD^TM^Lite application. A dietitian contacted the participants the following day to collect the 24 h recall data. The collected images and 24 h recall were used to retrospectively assess the goFOOD^TM^ system accuracy in estimating the energy and macronutrient intake of each participant. We further note here the difference between the goFOOD^TM^Lite application and the goFOOD^TM^ system. The former is used in this study solely for data collection, while the latter receives as input one or two images (extracted from the video recorded from goFOOD^TM^Lite). Even though, the goFOOD^TM^ system needs one or two images to perform automatic dietary assessment, we still asked the users to capture a short video in case the images would be blurry.

### 2.1. goFOOD^TM^Lite Application

**Figure 1:**
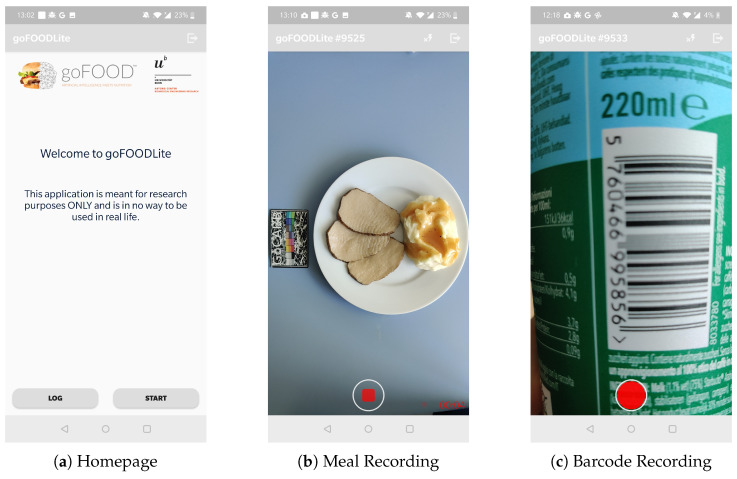
Screenshots of the goFOOD^TM^Lite application.

goFOOD^TM^Lite is an Android application used for recording meals during the initial phase of our feasibility study. This application allows participants to record meals, including food items, drinks, and packaged products, without receiving feedback on nutritional content. For food and drinks, recordings were logged in the form of a short video (∼10 s), while for packaged products, an image of the product’s barcode had to be recorded. Particularly for barcode and food logging, users have to choose the label of either snack, breakfast, lunch, or dinner. To enable reliable retrospective analyses, users have to take videos both before and after consumption. In the case of barcodes, the user also has to indicate the percentage of the packaged product that was consumed. Users could also consult their previous recordings in the history log screen. Figure 1 shows the application’s homepage, meal recording, and barcode recording screens.

### 2.2. Feasibility Study

To further optimise, extend, and improve the goFOOD^TM^ system [27], we recruited 50 adult participants across Switzerland who were proficient in German or English and could use an Android smartphone. Individuals adhering to specific diets, studying nutrition/dietetics, or dealing with neurocognitive or terminal-stage diseases, were excluded from the study in order to maintain homogeneity and avoid potential biases. The participants were recruited through various methods, including our website, social media advertisements, and university mailing lists. Once participants had been screened and expressed their willingness to participate in the study, they were required to fill out a demographics questionnaire and sign a consent form. Participants then had to attend an online session to be instructed on how to use the goFOOD^TM^Lite application, which was supported by information on the study. We then provided the participants with an Android smartphone with the app installed, along with a reference card that would be used to analyse the collected data retrospectively.

For a single day, the participants had to record their food and drink items before and after consumption, while ensuring that the reference card was placed next to the meal items. To retrospectively analyse the data collected and evaluate our system, we extracted two images from the videos at 90° and 75°, as based on the smartphone orientation. In the case of the consumption of a packaged product, the users had the option of capturing a photo of the product’s barcode.

In addition, users had to fill out a feedback questionnaire regarding the goFOOD^TM^Lite app. We utilised a feedback questionnaire based on the validated System Usability Scale questionnaire [30], as well as incorporated additional questions tailored for the usage of goFOOD^TM^Lite. Specifically, the user had to grade from a scale of 1–5 (very bad, bad, neutral, good, very good, respectively) their satisfaction regarding the following elements: (1) recording/logging, (2) time needed, (3) usability/user interface, (4) functionality/performance, and (5) user satisfaction/usage. The participants were also asked if they would be willing to use the app in their everyday life and if they would recommend it to friends. Finally, they were asked about what features they liked and disliked, and if they would prefer for the app to offer food content estimation based on the images. Both the demographics and the feedback questionnaires were created and handed out to the participants via the REDCap web application [31].

The following day, a dietitian contacted the participants and asked them about all of the foods and beverages they had consumed over the previous 24 h with the Multiple-Pass Method [32]. The dietitians used the nut.s nutritional software [33] and the United States Department of Agriculture [34] and Swiss nutrient database [35] for analysing the nutrients.

### 2.3. Database

The images collected from the feasibility study comprise the Swiss Real Life 2022 dataset (SwissReLi2022). In particular, SwissReLi2022 contains 444 images with 587 food items. We annotated the images using a semi-automatic tool and only used those that were taken before consumption, keeping one for each meal. Labels were assigned based on the food categories supported by our system. For ease of understanding and categorisation, we split the different foods into 18 coarse, 34 middle, and 301 fine categories (e.g., meat/red meat/meatball). This categorisation was based on our previous dataset [27], and from the food categories collected from the images during the feasibility study. To evaluate the full system, as described in Section 2.4.1, we filtered out the erroneous images, e.g., those from the excluded participants or duplicates, and ended up with 548 recordings, including 55 barcode images. Of these, 47, 61, 64, 81, and 295 corresponded to breakfast, lunch, dinner, snack, and drinks (of which 195 were water), respectively.

As previously mentioned, participants were instructed to capture images of their meals both before and after their meals. While this was true for most cases, a few participants did not record their post-consumption. Moreover, when these post-consumption recordings were available, we observed empty plates or dishes, obviating the necessity to calculate calories for these images. Consequently, only the pre-consumption recordings were taken into account.

For the segmentation task, we compiled a database consisting of previously collected images [29,36,37] and v2.1 of the publicly available MyFoodRepo dataset [38]. The segmentation dataset contained approximately 57,000 images with 107,000 segments. We used the SwissReLi2022 dataset to evaluate the segmentation module and 120 pictures from the MADiMa dataset, as captured from different angles [37].

For the classification task, we used web-crawled data and images from MyFoodRepo [38]. We ended up with approximately 200,000 images, which were divided into the categories supported by our system as previously mentioned. To test the recognition module, we used the 587 annotated items from the SwissReLi2022 and 234 items from the MADiMa dataset [37].

### 2.4. Automatic Dietary Assessment

#### 2.4.1. System Pipeline

**Figure 2:**
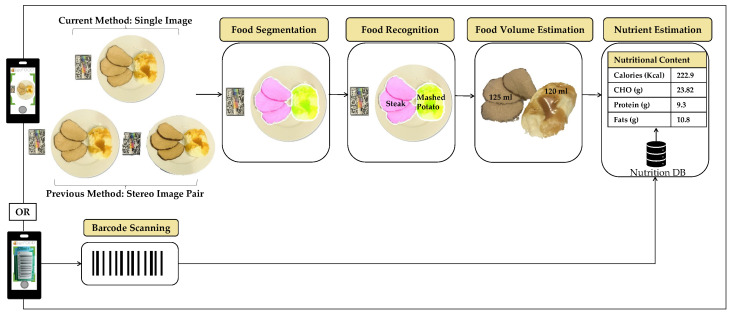
The goFOOD^TM^ system pipeline. The previous version of our system required two images from different angles as input, while the new method requires only a single image.

The goFOOD^TM^ system is designed to offer a complete automated pipeline for dietary assessment. It consists of five modules, as illustrated in Figure 2: (1) food segmentation, (2) food recognition, (3) food volume estimation, (4) barcode scanning, and (5) nutrient estimation. In the previous implementation of the goFOOD^TM^ system, two images were required as input, while the new adapted method needs only a single image as its input. The images, which are extracted from the video, are sequentially processed by a segmentation and recognition network. Afterwards, the module for estimating the food volume generates a 3-D model and makes an accurate prediction of the volume of each item. Finally, the module for nutrient estimation provides information on the kilocalories (kcal) and macro-nutrient content of the entire meal (as well as for barcode food products), based on existing databases. In Figure 2, we show both the current and the previous goFOOD^TM^ system pipeline with one or two images, respectively.

#### 2.4.2. Food Segmentation

Given the substantial variations in food datasets in terms of classes, we chose to train the segmentation network exclusively on food-versus-background data. This approach allowed us to merge diverse datasets for food-versus-background segmentation. Subsequently, the recognition task was handled by a separate model, eliminating the need to find a dataset that includes both segmentation masks and specific classes. As detailed in Section 3.2.1, our decision proved to be more effective compared to the alternative of combining segmentation and recognition into a single model. By focusing on binary segmentation first and then employing a separate recognition module, we achieved superior results in our overall system.

The first module of the pipeline comprises a Convolutional Neural Network (CNN) to segment the food items within the picture. We used a state-of-the-art segmentation network, namely Mask R-CNN [39], which was pre-trained on the COCO dataset [40]. As the backbone, we adopted the popular ResNet-50 [41], which was pre-trained on ImageNet [42]. We set the batch size to 8, and used the Adam optimiser with a learning rate of $10^{-4}$ and a weight decay of $5\times 10^{-4}$ for six epochs.

#### 2.4.3. Food Recognition

Each segmented item from the previous task was fed into a recognition network that predicted categories at three different levels of granularity: coarse, middle, and fine. We selected RegNetY-16GF as the classification network [43], i.e., a CNN trained through neural architecture search to find the optimal parameters (e.g., block width and network depth).

To address the label noise present in the dataset for training and to ensure accurate predictions, we adopted a noise-robust training approach. We utilised DivideMix [44], which has proven to be effective in handling label noise—even in the context of food images [22]. In particular, DivideMix trains two networks for a few epochs as a warmup to prevent overfitting to the label noise. Then, the two networks separate the training set into a clean and a noisy subset. The labels of the clean subset are further refined based on their probability of being clean and the network’s predicted labels. On the contrary, the noisy subset’s labels are substituted by the average of both networks’ predictions.The MixUp [45] technique then interpolates the samples in the training set so that the model learns to make linear predictions on the linear interpolations of the images.

Input images were resized to 256×256, randomly horizontally flipped, and then normalised. We followed the DivideMix [44] training pipeline and set the number of warm-up epochs to three and subsequent training for another five; this was achieved using the Adam optimiser with a batch size of 32 and a learning rate of $10^{-4}$.

#### 2.4.4. Food Volume Estimation

The module for the estimation of the food volume necessitated translating 2-D food items into a 3-D space. To facilitate this process, it was essential to position a reference card with known dimensions within the field of view, as shown in Figure 2. In our study, we utilised two approaches to generate depth maps, which served as the initial step in converting food items into 3-D. The first approach involved a neural-based approach using a single image. In contrast, the second approach utilised a geometry-based approach using image pairs, as outlined in [27]. Next, the known distance between the reference card and the camera enabled the translation of the depth map to the actual world distances. We implemented a filtering process to remove noise from the depth map, and thus ensured that the information on depth was accurate and reliable. The segmentation mask, obtained from the segmentation module, was applied to the depth map; this allowed us to associate each depth value with the corresponding food item, effectively separating the different objects in the scene. Based on the segmented depth map, each food item was re-projected to a 3-D point cloud, cleaned through outlier removal whenever necessary, and scaled to real-world coordinates based on the reference card. We computed the volume of each food item within this transformed space, thus enabling accurate estimation of the food volume. In cases where the reference card was absent or undetected, we used the volume of a standard serving of the food item, as both volume estimation methods rely primarily on the presence of the reference card. Moreover, in order to remove noise within the 3-D point clouds, we assumed that no food item could have a volume of over 2.5 cups.

#### Neural-Based Approach

The main contribution of the enhanced version of goFOOD^TM^ is the usage of single images, for depth estimation, taken at 90°. For this purpose, we employed a model to estimate depth, namely Zoe [46], which combines multiple depth modules within an encoder–decoder architecture. Zoe was a suitable candidate for our depth estimation module since it was trained on diverse indoor datasets and demonstrated robust generalisation performance. To further improve the prediction accuracy, we also introduced certain rules to prevent significant misestimations originating from the model itself or due to poor quality images. In particular, after predicting the depth map and converting it into real-world distance values, we made two reasonable assumptions to remove possible outliers. Firstly, we specified that the bottom of the food item plane should not be excessively distant from the card, with a fixed maximum of 5 mm. Secondly, we set a constraint for the top part of the food item, based on our data collected, within the 3D model, and thus ensured that it did not extend more than 5 cm above the card plane.

#### Geometry-Based Approach

The earlier goFOOD^TM^ version used a stereo matching pipeline, which entailed capturing two images of the meal at two angles (90° and 75°) [27]. In this work, we slightly refined some components to overcome a key limitation of the previous food volume estimation module—the need for food to be placed on a plate. In practice, we first detected key points from the reference card and the generated segmentation masks for each image. The stereo image pair was rectified, thus aligning points in one image with the corresponding row in the second image while correcting distortions. Following the rectification process, a disparity map was created by horizontally comparing the differences between the pixels from the first and second images in order to infer depth information about the scene. Subsequently, the disparity was converted into a depth map.

#### 2.4.5. Nutrient Estimation

Once we had obtained the volume for each food item, we automatically fetched its nutritional content from nutrient content databases. Despite dietitians utilising the USDA and Swiss food databases, there was a lack of clarity regarding the specific database employed for each food item. As our major evaluation criterion relies on volume rather than on weight, which is not provided by the USDA database directly, we utilised Nutritionix [47] and AquaCalc [48], which both rely on the USDA database. While Nutritionix contains most food item information and volume-to-weight ratios, in the event there was a missing food item, we used AquaCalc.

The final output was the nutritional content of the full meal. The selection of the database was primarily aimed at improving data standardisation. Additionally, the nutritional information of packaged products was extracted from the Open Food Facts database [49].

## 3. Results

### 3.1. Feasibility Study

Of the 50 participants, 35 were female, and most of them were of a white ethnicity (90%). The volunteers were generally young, with an average age of 29.2 years and a standard deviation of 11.4 years; moreover, the majority were students (27/50). Table 1 shows the demographics of the 50 recruited participants. One participant was excluded from the analysis since their 24 h recall was not completed.

**Figure 3:**
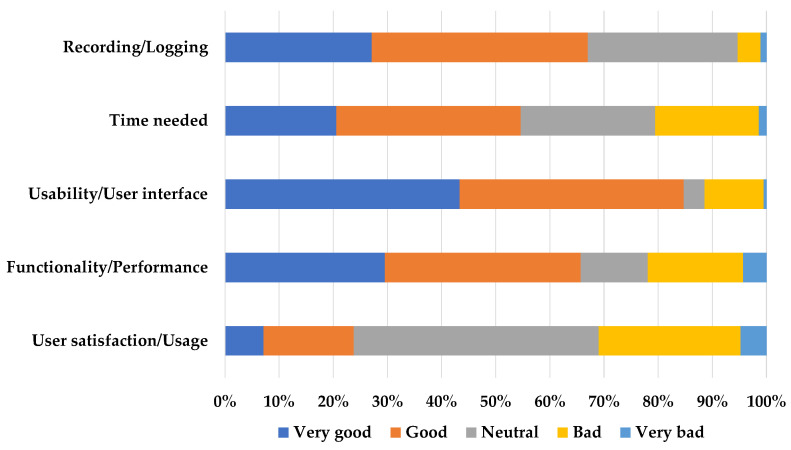
User feedback questionnaire.

Of the 50 participants enrolled in the study, 8 did not complete the final feedback questionnaire. Based on the responses, 29/42 of the participants (69%) agreed that the logging and recording were good or very good, while 35 (83.3%) found the app easy to use and self-explanatory. Regarding the time needed to complete a recording, 23/42 (54.8%) agreed it was good or very good, while 10 (23.8%) expressed no opinion. However, when the participants were asked, in the “user satisfaction/usage” question, if they would use the app, 19 (45.2%) remained neutral. On the other hand, 22/42 (52.4%) participants said that they would be willing to use the goFOOD^TM^Lite application for tracking their food intake in their everyday life, while 30 (71.4%) answered that they would recommend it to their friends. Lastly, almost all the participants (41/42) answered positively when asked if they would like the application to offer automatic food content estimation, and 28 said they would be willing to participate in the upcoming validation study. The participants’ answers to the feedback questionnaires are visualised in Figure 3.

### 3.2. Automatic Dietary Assessment

#### 3.2.1. Food Segmentation and Recognition Evaluation

**Figure 4:**
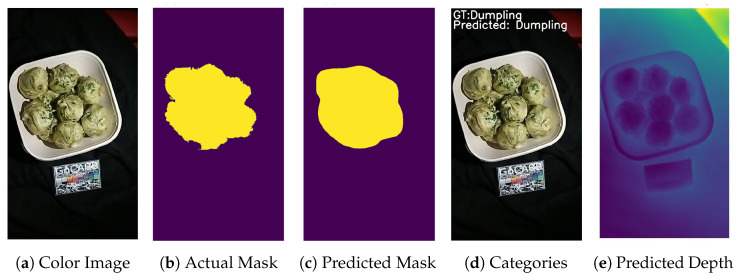
A sample image from the SwissReLi2022 dataset. (**a**) The colour image. (**b**) The GT segmentation mask. (**c**) The predicted mask by the model. (**d**) The GT and predicted categories. (**e**) The predicted depth by the model.

Initially, we evaluated the Mask R-CNN instance segmentation network trained on the binary segmentation task (food-vs.-background). We additionally trained a Mask R-CNN that predicted not only the food items’ positions, but also their coarse class (meat products, cereals/potatoes, liquids, dessert, and fruits/vegetables/nuts). The binary segmentation network achieved an intersection over union (IoU) of 74%, thus outperforming the other model (which scored 67% IoU). We argue that the reason for this is that the binary model is less prone to overfitting, since it only has to discriminate between two macro-classes. Figure 4 shows the color image (a), the GT mask (b), and the mask predicted by the Mask R-CNN (c) for one meal.

We also evaluated the recognition CNN using the GT annotated food items as input. As metrics, we used the top-1 and top-3 accuracy for the coarse and middle categories, and the top-1 and top-5 accuracy for the fine food categories. We compared the ResNet-50 model and the RegNetY-16GF with or without the DivideMix training procedure to contrast the label noise. The results are shown in Table 2. RegNetY-16GF with the DivideMix procedure clearly outperformed the other approaches for the fine-grained recognition task, which is the most critical since the nutritional values are based on the fine categories.

#### 3.2.2. System Results

Within our study, we designed a comprehensive pipeline for dietary assessment that encompassed multiple stages and algorithms, as previously mentioned, to estimate dietary intake. To establish a reliable benchmark, we relied on the expertise of professional dietitians and their 24 h recall data, which we considered as the baseline for comparison in the absence of GT weights and volume. However, it is important to note that misestimations from dietitians can be quite high. For instance, as shown in a previous study, the mean absolute error for the carbohydrate (CHO) estimations of dietitians reached 15 g [50]. The dietitians performed a dietary intake assessment for each participant for the whole day, but not for every meal. Therefore, we evaluated the performance of our system for each participant individually.

Quantifying the accuracy and variability of the system’s estimations was vital in understanding the overall performance. We compared the results obtained when using the two approaches (as shown in Table 3): using a single image for our new method, and using two images for the previous method. We further assume that if the GT food category was among the top five predicted categories, the user would select it and the GT class would be chosen. We assessed the percentage error in terms of four key dietary components: kcal, CHO, protein, and fat. We considered the evaluation of kcal as the most significant, given its applicability for everyday use. Since our analysis considered the entire day’s intake rather than individual meals, there was no necessity to separate between results for packaged and non-packaged products within this study. For the newly proposed method that uses a single image, the mean absolute percentage error in kcal estimation per participant was 27.41%. Furthermore, we observed a percentage error of 31.27% for the CHO, 39.17% for the protein, and 43.24% for the fat estimation compared to the dietitians’ estimations, which used the 24 h recall method. The previous method that used two images, gave a percentage of error of 31.32% for the kcal, 37.84% for the CHO, 42.41% for the protein, and 51.75% for the fat intake estimations.

**Figure 5:**
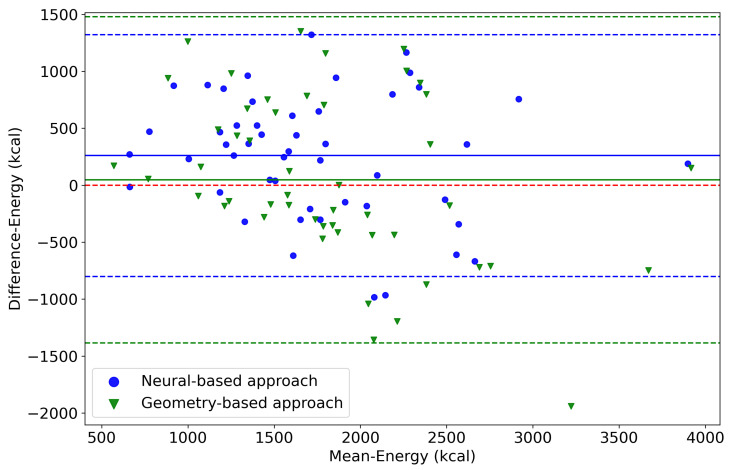
Bland–Altman plot of the neural-based approach (blue) and the geometry-based approach (green) in terms of energy (kcal) versus the dietitians’ estimations, which used the 24 h recall method. The dashed blue and green lines indicate the 95% confidence intervals, while the continuous line is the mean difference. The dashed red line indicates zero difference between the dietitian and the goFOOD^TM^ approaches.

Even though the new method achieves a slightly lower empirical mean absolute percentage error, the results did not exhibit a statistically significant difference under an independent t-test. Nevertheless, the new method only requires a single image as input, whereas the previous method uses two images from specific angles. Our lowest misestimation percentages with the new method for kcal, CHO, protein, and fat were 2.16%, 0.34%, 3.46%, and 0.02%, respectively. Figure 5 shows the Bland–Altman plot for the 49 participants regarding their energy intake (kcal) for one day. The estimations of the neural- and the geometry-based approach appear in blue and green, respectively. The plot shows that although both methods tended to overestimate the kcal compared to the dietitians, the geometry-based approach had a higher variance.

## 4. Discussion

We conducted a feasibility study in Switzerland to assess the user satisfaction with the goFOOD^TM^Lite application, as well as collected data for a retrospective analysis of individual dietary habits. The participants found the app easy to use, and most of them would recommend it to their friends. It is worth noting that 41 of 42 participants said they would like to receive nutrient information for their meals. However, a significant proportion of participants were female and students of white ethnicity. We note that to ensure more robust and comprehensive conclusions, it would be essential to incorporate a broader and more diverse population.

We compared the performance of two methods in order to evaluate their suitability: a neural-based depth estimation method that uses a single image as input, and a geometry-based approach that uses two images. The newly introduced method utilised a single image as input and achieved similar results to our previous approach regarding energy and macronutrient intake. However, the new method can potentially enhance user satisfaction and adherence to the system by requiring only a single image capture. User preference plays a pivotal role in the adoption and success of any system. Our previous study demonstrated that users prioritise systems that minimise effort, even if this forfeits accuracy [28].

During this study, we encountered several challenges that affected the overall performance and reliability of the system. The first challenge was related to meal recording. The acquisition of two images from a video, based on the smartphone’s position, can result in occasional blurry pictures due to user movements and variations in lighting conditions. Using different phones with varying camera resolutions led to additional variability in image quality. Moreover, discrepancies were observed in individuals’ compliance in recording their meals, with both meal omissions, multiple entries, or neglecting the use of the reference card. The person with the highest error for the kcal intake (64.53%) recorded only four meals, of which one depicted a glass of water, and in two, the reference card was missing. These issues could potentially introduce inconsistencies and inaccuracies in our results.

Another challenge stems from the fact that the system does not account for user-specific information. For both approaches, the system exhibited the highest misestimation in the case of fat. This can be attributed mainly to the amount and type of oil and butter present in the food, which varies for each participant and cannot be visually extracted from images. For example, the participant with the highest error in kcal estimation used 50 g of olive oil, which leads to an additional 400 kcal and 45 g of fat. It is important to note that not only do ingredients affect the nutritional content of foods, but different cooking methods also play a role in this matter [51]. It is crucial to acknowledge that even though our system supports mixed and layered food items (e.g., rice soup and lasagna), the way meals are prepared and presented might affect their complexity and, hence, the precision of our system. In the future, approaches such as incorporating recipes or manual entries could be introduced in the pipeline to tackle these issues.

Furthermore, the absence of GT exacerbates the complexity of this study. Without reliable reference measurements for food volumes, it is challenging to provide an objective assessment of the accuracy and validity of our estimation methods. Although 24 h recalls are commonly used, they can introduce major errors in energy and macronutrient information, as they rely on a person’s memory and subjective estimation of portion sizes [52]. Additionally, in the volume estimation module, the formulation of the depth map incorporates inherent assumptions as part of the established methodology. Estimating accurate depth maps and evaluating them is challenging without a depth sensor.

To address the challenges encountered in this study, as well as to further improve and optimise the system to meet the end user expectations, a second phase will take place. In this next phase, the involved participants will be asked to use the goFOOD^TM^ system in real-time for one week, as well as record their food intake with an FFQ and participate in two unannounced 24 h recalls. Users will directly capture pictures from the goFOOD^TM^ app, thus eliminating the use of video recording methods. The users will see the results of the segmentation, recognition, and volume estimation modules, and will be able to change them if needed. The users will also have the option to manually input the amount of hidden ingredients, like oil or butter, used for a meal. Through app nudges, we aim to encourage users not to neglect their meals and actively utilise the reference card. In future investigations, integrating a food weighing method or submitting captured images to a qualified dietitian for meticulous assessment emerges as the most promising alternative to dietitians performing 24 h recall. Finally, we must acknowledge the need to refine our system to ensure its effectiveness across various food types and under multiple conditions, such as varying distances and viewing angles. The depth model we used was not specifically trained on closely viewed items; therefore, its effectiveness in accurately estimating volumes for such items may require additional optimisation. These adjustments will contribute to the enhanced accuracy and dependability of the collected data, as well as ultimately improving the performance of our system.

## 5. Conclusions

This manuscript presents a fully automatic system that estimates energy and macronutrients from a single meal image. The newly introduced method within the system demonstrates a comparable performance, but the neural-based approach is more user-friendly as it only requires a single image. This indicates that our system has the potential to facilitate the monitoring of individuals’ dietary habits while reducing the costs associated with dietary assessment. However, further improvements are needed to ensure that the system is effective for closely viewed items, as well as to address the challenges related to depth map formulation, image acquisition, and user compliance. For future work, we plan to conduct the second phase of this study, which will involve participants using the goFOOD^TM^ system directly on their smartphones for a longer period. Thus, we will obtain real-life data, which will be used to validate and refine the system’s accuracy and reliability.

## Figures and Tables

**Table 1:** Participant Demographics (*n* = 50, %).

Characteristic	Value
Sex (*n*, %)	Female (*n* = 35, 70%)
	Male (*n* = 15, 30%)
Mean age in years (SD)	29.2 (11.4)
Mean BMI in kg/m^2^ (SD)	27.2 (11.8)
Ethnicity (*n*, %)	White (*n* = 45, 90%)
	Hispanic/Latino (*n* = 2, 4%)
	Asian/Pacific Islander (*n* = 2, 4%)
	Half European/Half Latino (*n* = 1, 2%)
Highest Educational Level (*n*, %)	High School/Apprenticeship (*n* = 16, 32%)
	Bachelor’s Degree (*n* = 19, 38%)
	Master’s Degree (*n* = 7, 14%)
	PhD (*n* = 3, 6%)
	Other (*n* = 5, 10 %)
Occupation (*n*, %)	Student (*n* = 27, 54%)
	Employed full time (*n* = 15, 30%)
	Employed part time (*n* = 5, 10%)
	Self-employed (*n* = 1, 2%)
	Retired (*n* = 1, 2%)
	Homemaker (*n* = 1, 2%)

**Table 2:** Comparison between the % accuracy of the different recognition models.

Models	Fine		Middle		Coarse	
	Top-1	Top-5	Top-1	Top-3	Top-1	Top-3
ResNet-50	52.9	67.1	68.2	85.3	75.3	88.8
RegNetY-16GF	56.4	70.6	74.1	86.8	80.3	91.1
ResNet-50 + DivideMix	55.4	66.5	70.6	85.0	75.9	88.1
RegNetY-16GF + DivideMix	58.7	78.8	75.0	89.0	79.3	91.1

**Table 3:** Mean absolute percentage error (%) between the two systems and the dietitians’ estimation for the energy and nutrient intake. In the parentheses, the standard deviation is shown.

Complete System	kcal	Carbohydrates	Protein	Fat
Geometry-based	31.32 (22.3)	37.84 (36.4)	42.41 (25.1)	51.75 (57.4)
Neural-based	27.41 (16.9)	31.27 (22.4)	39.17 (33.9)	43.24 (32.4)

## Data Availability

The datasets generated and/or analysed during the current study are not publicly available due to privacy constraints, but are available from the corresponding authors on reasonable request.

## Abbreviations

The following abbreviations were used in this manuscript:

AI	Artificial Intelligence
CHO	Carbohydrates
CNN	Convolutional Neural Network
FFQ	Food Frequency Questionnaire
GT	Ground Truth
IoU	Intersection over Union
kcal	Kilocalories
